# Imperforate Anus With Perineal Fistula in an Infant Presenting With Constipation and Abscesses: A Case Report

**DOI:** 10.7759/cureus.95466

**Published:** 2025-10-26

**Authors:** Amia Mourad, Krishna Patel, Nicholas Lorenz, Hibah Mohammed, Madhura Butala

**Affiliations:** 1 Medicine, Lake Erie College of Osteopathic Medicine, Bradenton, USA; 2 Pediatrics, Ascension St. Vincent's, Jacksonville, USA

**Keywords:** anorectal malformation, bowel continence, colostomy, constipation, fecal impaction, imperforate anus, pediatric surgery, perianal abscess, perineal fistula, posterior sagittal anorectoplasty

## Abstract

Chronic constipation and failure to thrive in infants can sometimes mask underlying congenital anorectal malformations. Here, we present a female infant who initially presented with refractory constipation, slow weight gain, and intermittent vomiting, later found to have an imperforate anus with a perineal fistula. Her course was complicated by recurrent fecal impactions, perianal and gluteal abscesses, and repeated hospitalizations. Multiple interventions were required, including manual disimpactions, rectal dilations, full-thickness biopsies, a colostomy with mucus fistula creation, a posterior sagittal anorectoplasty (PSARP), anal Botox-assisted dilations, and an eventual colostomy takedown. Her structural anorectal abnormality led to chronic stool retention, creating a nidus for bacterial overgrowth and tissue breakdown, which explained the recurrent perianal abscesses. Despite early feeding difficulties, recurrent impactions, and postoperative challenges, by age three, she achieved near-complete bowel continence, age-appropriate growth, and developmental milestones. This case illustrates the complexity of managing anorectal malformations and emphasizes the importance of staged interventions, multidisciplinary care, and structured bowel regimens in achieving long-term functional outcomes.

## Introduction

Anorectal malformations are congenital anomalies of the distal hindgut that arise from the abnormal development of the cloacal membrane and urorectal septum during embryogenesis [1]. They occur in approximately one in 4,000-5,000 live births and encompass a wide spectrum, ranging from mild anal stenosis to complex cloacal malformations [2]. Low-type defects, such as imperforate anus with a perineal fistula, often result in a narrowed or ectopic anal opening that permits partial stool passage, potentially obscuring early recognition.

The pathophysiology of anorectal malformations is closely linked to the impaired evacuation of stool through the abnormal outlet. Obstruction and incomplete emptying lead to chronic fecal retention, distal colonic dilation, and increased intraluminal pressure [3]. This stasis favors bacterial overgrowth, mucosal injury, and inflammation, while repeated straining or instrumentation can cause further local trauma. Over time, these mechanisms contribute to complications such as recurrent impactions, perianal infections, and poor growth [4]. These pathophysiologic mechanisms and their clinical consequences are illustrated below.

Although early signs may be mistaken for functional constipation or Hirschsprung disease, the underlying structural abnormality requires surgical correction to restore anatomy and preserve continence potential [5]. Recent advances in imaging have improved the early detection and anatomical characterization of anorectal malformations. Pelvic ultrasound and magnetic resonance imaging (MRI) can help delineate the relationship between the rectum, sphincter complex, and adjacent pelvic structures and may aid in diagnosis and surgical planning even in subtle or low-type variants [6,7]. However, despite these technological advancements, establishing a definitive diagnosis can remain challenging, particularly in cases with atypical or mild presentations. The following case highlights how a delayed diagnosis of imperforate anus with a perineal fistula led to recurrent abscesses and hospitalizations and how a staged multidisciplinary approach ultimately enabled favorable long-term outcomes.

## Case presentation

The patient was a full-term female infant with normal initial bowel movements and appropriate birth weight. At five months, she presented with constipation, slow weight gain, and possible gastroesophageal reflux. At this visit, the pediatrician discussed starting solid feeds and supplementing with formula. By eight months, she developed fecal impaction, abdominal distension, and minimal stool output, prompting the initiation of laxatives and referral to a gastroenterologist. She was advised to continue the EleCare formula and introduce naturally laxative fruits. Early perineal findings were not detailed in the documentation.

**Figure 1:**
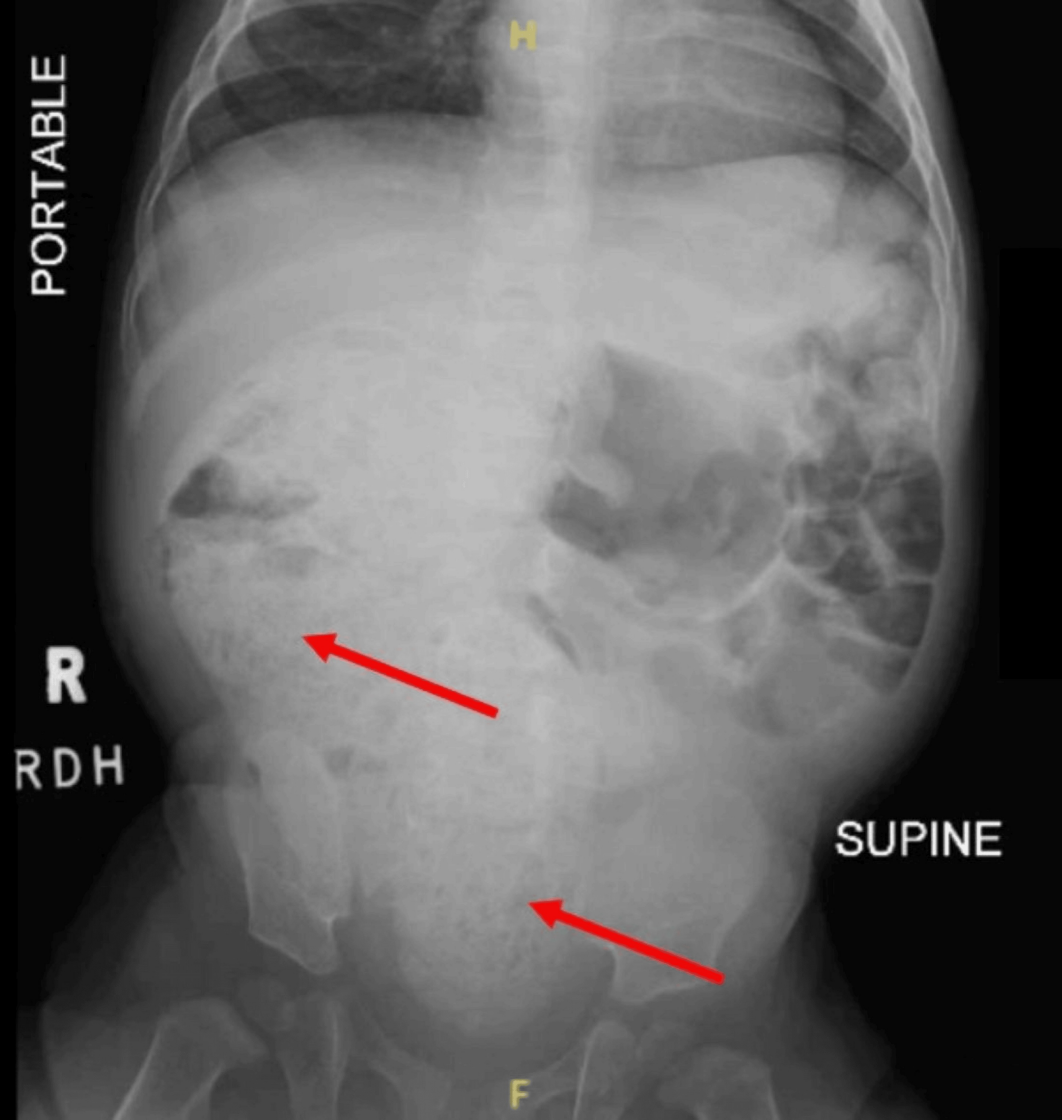
Abdominal radiograph demonstrating a non-obstructive bowel gas pattern with a large amount of stool in the right abdomen and pelvis (arrows)

At nine months, urgent care evaluation revealed severe constipation with abdominal tenderness and palpable stool masses, and imaging confirmed a significant fecal burden (Figure 1). She was given a glycerin suppository, and at the pediatrician follow-up the next day, a regimen of glycerin suppository pediatric size, one per day for three days until daily stools, and milk of magnesia 2.5 mL at bedtime for 15 days was started. Constipation initially improved with the suppository but recurred once it was discontinued. Rectal examination noted hard stool within the rectal vault, but the position and appearance of the anal opening were not documented.

**Figure 2:**
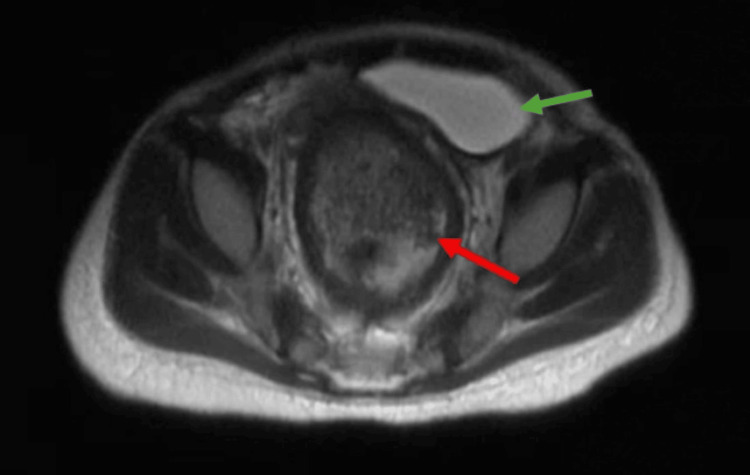
Axial pelvic magnetic resonance imaging (MRI) demonstrating marked rectosigmoid stool burden (red arrow) causing anterior and leftward displacement of the urinary bladder (green arrow)

At 11 months, she was admitted for the evaluation of suspected Hirschsprung disease. Full-thickness rectal biopsies were inconclusive. During hospitalization, she developed recurrent bilateral gluteal abscesses requiring incision, drainage, and intravenous antibiotics. Manual disimpactions and rectal dilations were necessary to relieve fecal impactions, and MRI demonstrated a marked rectosigmoid stool burden with bowel wall thickening (Figure 2). Persistent constipation and repeated perianal infections highlighted the need for structural evaluation. 

**Figure 3:**
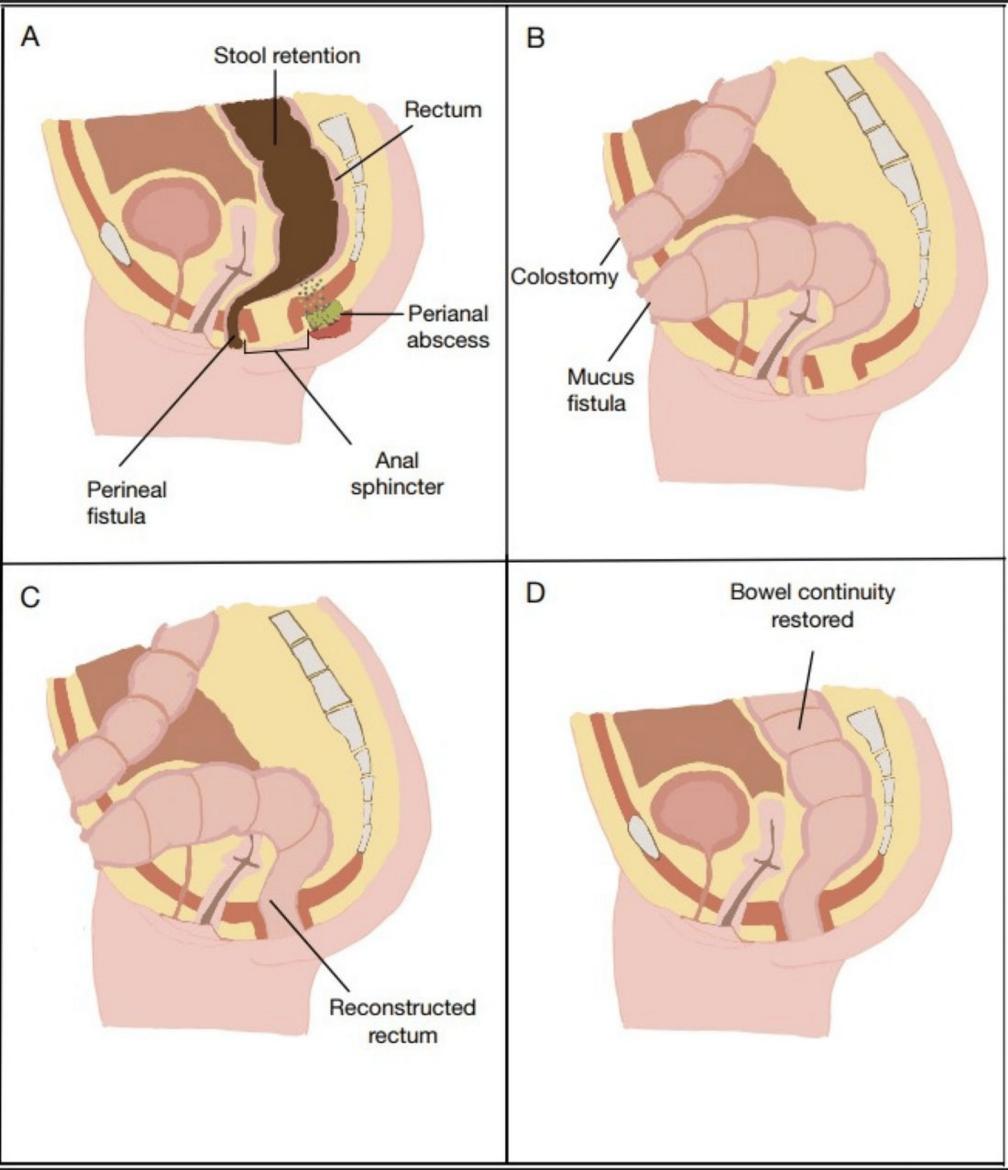
Clinical course of imperforate anus with perineal fistula. (A) Distal rectum ending in perineal fistula with stool retention and perianal abscesses. (B) Diverting colostomy with mucus fistula. (C) Posterior sagittal anorectoplasty (PSARP) with the rectum positioned through the sphincter complex. (D) Colostomy takedown with restored bowel continuity Original illustration by Amia Mourad

At 12 months, her pediatrician recommended continuation of toddler formula and addition of prune juice if stools became hard. At 13 months, rectal examination under anesthesia confirmed an imperforate anus with a perineal fistula (Figure 3A). Laparoscopic colostomy with mucus fistula creation was subsequently performed (Figure 3B). Histopathology confirmed normally ganglionated colorectal tissue, ruling out Hirschsprung disease [8]. Postoperatively, she tolerated pureed feeds, and the colostomy functioned appropriately. Parents were instructed on bowel management, including rectal dilations twice daily and a regimen of Senna and lactulose.

Over the following year, she underwent a posterior sagittal anorectoplasty (PSARP) with postoperative anal dilations and anal Botox injections to relax the sphincter, which facilitated gradual dilator progression and helped prevent strictures [9] (Figure 3C). This was followed by a colostomy takedown at two years old, with continued anal Botox injections (Figure 3D). Postoperative care included structured bowel regimens with MiraLAX and as-needed Senna, which helped maintain regular soft bowel movements. Despite intermittent constipation that required cleanouts, she progressed to having daily stools and improved stool consistency.

By three years of age, the patient achieved near-complete bowel continence, with one soft bowel movement daily and minimal use of laxatives as needed. She demonstrated approximately 90% success in age-appropriate toilet training with no rectal prolapse, recurrent infections, or other complications. Growth and developmental milestones were on track. 

## Discussion

Low-type anorectal malformations can be difficult to detect in infancy, particularly when partial stool passage is possible through a narrow perineal opening. In this patient, early symptoms were attributed to functional constipation, and initial evaluations, including rectal biopsies, were inconclusive. The pelvic MRI did not clearly identify the location of the ectopic anal opening likely due to the narrow perineal fistula and the subtle nature of low-type anorectal malformations on imaging. In infants with low-type anomalies, a small anteriorly displaced anal opening may mimic a normal anus, further complicating early recognition [10]. The combination of diagnostic challenges and atypical anatomy contributed to prolonged fecal retention, recurrent impactions, perianal and gluteal abscesses, and repeated hospitalizations. A meticulous perineal examination during early infancy may have facilitated earlier diagnosis, potentially allowing for a single-stage corrective procedure rather than a multi-stage approach. These complications highlight both the clinical impact of unrecognized structural disease and the importance of vigilance for subtle anomalies in similar presentations [11].

Management required a carefully staged approach. Diversion with a colostomy allowed the decompression and resolution of infection while improving her nutritional status. PSARP restored the alignment of the rectum within the sphincter complex, and adjunctive therapies such as anal dilations and Botox injections minimized stricture formation. Postoperative bowel regimens further optimized stool consistency and regularity, helping preserve continence potential. Growth and developmental milestones remain on track, illustrating the long-term functional benefits of timely surgical and postoperative management even after delayed diagnosis [12].

Similar presentations of perineal fistulas and low-type anorectal malformations have been reported in the literature, emphasizing the diversity of anatomical variation and the critical role of thorough preoperative evaluation. For instance, Li and Wang describe a rare variant, a "long perineal fistula", in which the proximal colon lies unusually high and stool is conveyed through a narrow, elongated tract. In their series of seven patients, this variant was associated with poorer postoperative bowel function and reduced sphincter pressures compared to classic perineal fistulas, highlighting the functional impact of such atypical anatomy [13]. They reinforce the value of comprehensive imaging and individualized anatomic assessment to preserve continence and ensure favorable functional outcomes.

## Conclusions

Anorectal malformations can present as persistent constipation with poor growth, often mimicking functional disorders or Hirschsprung disease. Recurrent fecal impactions and perianal abscesses should prompt evaluation for structural anomalies. With timely recognition, staged surgical repair, adjunctive measures to optimize bowel function, and meticulous postoperative care, children with imperforate anus and a perineal fistula can achieve excellent functional outcomes. These outcomes include near-complete continence and normal development.
